# (-)-*O*-Methylcubebin from *Vitex trifolia* Enhanced Adipogenesis in 3T3-L1 Cells via the Inhibition of ERK1/2 and p38MAPK Phosphorylation

**DOI:** 10.3390/molecules25010073

**Published:** 2019-12-24

**Authors:** Motohiko Ukiya, Daisuke Sato, Hirokazu Kimura, Mamoru Koketsu, Nyunt Phay, Atsuyoshi Nishina

**Affiliations:** 1 College of Science and Technology, Nihon University, 1-5-1 Kandasurugadai, Chiyoda, Tokyo 101-0062, Japan; ukiya.motohiko@nihon-u.ac.jp; 2 Department of Biomedical Information Engineering, Graduate School of Medical Science, Yamagata University, 2-2-2 Iidanishi, Yamagata 990-9585, Japan; d_sato@yz.yamagata-u.ac.jp; 3 School of Medical Technology, Faculty of Health Science, Gunma Paz University, 1-7-1 Tonyamachi, Takasaki, Gunma 370-0006, Japan; h-kimura@paz.ac.jp; 4 Department of Chemistry and Biomolecular Science, Faculty of Engineering, Gifu University, 1-1 Yanagido, Gifu 501-1193, Japan; koketsu@gifu-u.ac.jp; 5 Botany Department, Pathein University, Main Rd, Pathein 10014, Myanmar; dr.nyuntpe@gmail.com

**Keywords:** *Vitex trifolia* L., adipogenesis, lipolysis, lipogenesis, rosiglitazone, (-)-*O*-methylcubebin

## Abstract

In this study, for the purpose of elucidation for antidiabetic components, we isolated and identified compounds that could become lead compounds for the development of antidiabetic agents from the herbal medicine *Vitex trifolia*, which is used for liver protection in Myanmar. Three kinds of lignan, (-)-*O*-methylcubebin (MC), (-)-hinokinin, and (-)-cubebin, were isolated from the ethyl acetate extract of the leaves of *V. trifolia*, using various chromatography. Among the three isolated compounds, MC showed the strongest effects to increase intracellular lipid accumulation in 3T3-L1 cells. From the results of the elucidation of the MC’s effects on the adipogenesis of 3T3-L1 cells, the downsizing of adipocytes and the promotion of the expression of adipogenesis-related proteins, as well as adiponectin, were observed. On the other hand, since the activity of MC was inhibited by antagonists of PPARγ and improved by inhibitors of the classical mitogen-activated protein kinase (MAPK) pathway and p38MAPK pathway, MC was considered to be an agonist of PPARγ, and furthermore promoted adipogenesis via the inhibition of extracellular signal-regulated kinase 1/2 (ERK1/2) and p38MAPK phosphorylation. Although MC showed similar effects to those of rosiglitazone (RO) used as a positive control, RO promoted the migration of GLUT4 from the cytoplasm to the cell membrane, whereas MC did not show such an effect. From the abovementioned results, it was considered that MC could be a lead compound for the development of antidiabetic drugs that does not show weight gain, which is a side effect of RO.

## 1. Introduction

Type 2 diabetes (T2D) affects more than 300 million people worldwide [1] and is one of the leading causes of death. The cause of T2D is a metabolic disorder caused by dyslipidemia, glucose intolerance, and inflammation. Peroxisome proliferator-activated receptor (PPAR) is a ligand-activated transcription factor that plays a crucial role in the regulation of glucose homeostasis and lipid metabolism, and it is considered to be one of the important targets for the treatment of metabolic disorders of T2D. To date, thiazolidinediones (TZDs), agonists of PPARγ, have been widely prescribed for the treatment of T2D. However, recent studies have shown that the use of these compounds is associated with undesirable effects such as weight gain [2] and an increased incidence of cardiovascular injury [3] and fractures [4]. Thus, the European Diabetes Association no longer recommends rosiglitazone, one of typical TZDs, for the treatment of T2D [5]. To avoid the side effects of TZDs, various strategies have been considered to develop compounds that act as ligands of PPARγ with fewer side effects. Clinical trials of a new class of TZDs, such as PPARα/γ dual agonists (Saroglitazar) and PPARpan agonists have been conducted but failed to avoid TZD-induced side effects because of their toxicity [6,7]. On the other hand, it has been reported that the modulation of PPARγ activity by selective PPAR modulators reduced insulin resistance and caused no side effects. [8,9]. Interestingly, several groups have shown that post-translational modification of PPARγ induced by different ligands affects the transcriptional effects on specific target genes, but an alternative medicine for TZD has not been developed yet [10,11,12]. On the other hand, many attempts have been reported to find ligands of PPARγ from natural medicinal plants having hepatoprotective effects [13,14].

*Vitex trifolia* L. (Lamiaceae) has been used as a hepatoprotective herbal medicine in Ayurveda (India), Unani (Islamic cultural zone), and Myanmar traditional herbal medicine. With regard to the biological activity of *V. trifolia*, its antibacterial activity has been reported [15,16,17]. It was found that flavonoids, diterpenes, benzofurans, etc. were contained in *V. trifolia* [18,19,20]. We previously reported that vitexylactone isolated from *V. trifolia* was a ligand for PPARγ and promoted adipogenesis [21]. Recently, three kinds of lignans were isolated and identified from the same plant, and all of the compounds promoted adipogenesis. In this report, we tried to clarify the action mechanism of adipogenesis by (-)-*O*-methylcubebin, which showed the most potent physiological activity among the three kinds of lignan analogues isolated from *V. trifolia* by comparison with the effects of rosiglitazone on adipogenesis.

## 2. Results

### 2.1. Regulatory Effects on Adipogenesis of the Extracts from V. trifolia

In order to isolate active components, hexane extract (7.8 g), ethyl acetate extract (12.2 g), and methanol extract (6.4 g) were obtained from 200 g of dried leaves of *V. trifolia*, as described in the previous report [21]. The hexane extract was found to be toxic at a concentration of 100 µg/mL, but the ethyl acetate extract and the methanol extract were not toxic at 100 µg/mL or less (see Supplementary Materials Figure S1(1)). On the other hand, regarding the results of the evaluation of the effects of these three extracts in 3T3-L1 cells during adipogenesis, the amount of intracellular lipid accumulation in adipocytes by the ethyl acetate extract was the highest (see Supplementary Materials Figure S1(2)), and from the results, we attempted to isolate and identify the bioactive components from the ethyl acetate extract. 

### 2.2. Characterization of the Isolated Compounds

**Figure 1:**
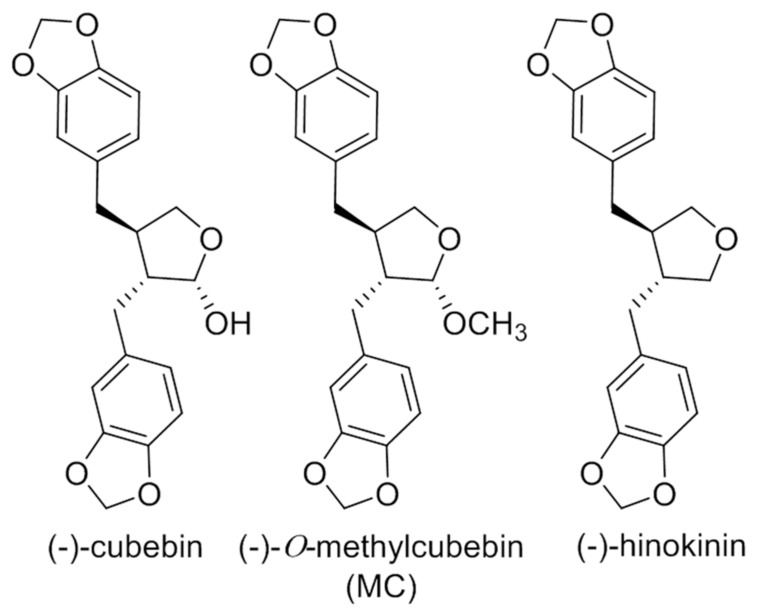
Isolated Compounds.

From the ethyl acetate extract, 3.9, 4.8, and 3.2 mg of compounds **1** [(-)-hinokinin], **2** [(-)-*O*-methylcubebin], and **3** [(-)-cubebin] were isolated, respectively (Figure 1). The structures of the isolated compounds were determined by spectral data. The molecular weight of compounds **1**–**3** was determined as 354, 370, and 370, respectively, by electron ionization mass spectrometry (EI-MS). Compound **1** displayed carbonyl characteristics in NMR and was identified as (-)-hinokinin [22]. Compound **2** was identified as (-)-*O*-methylcubebin (MC) previously isolated from *Artemisia chamaemelifolia* [23]. The NMR data of compound **3** possessed the characteristics of a lignan, and the structure was found to be (-)-cubebin [24] (^13^C-NMR data were shown in the Supplementary Materials, Table S4). The stereo structures of compounds **1** and **2** were determined by single-crystal X-ray analysis. 

### 2.3. Cytotoxicity of the Three Isolated Compounds in 3T3-L1 Cells

**Figure 2:**
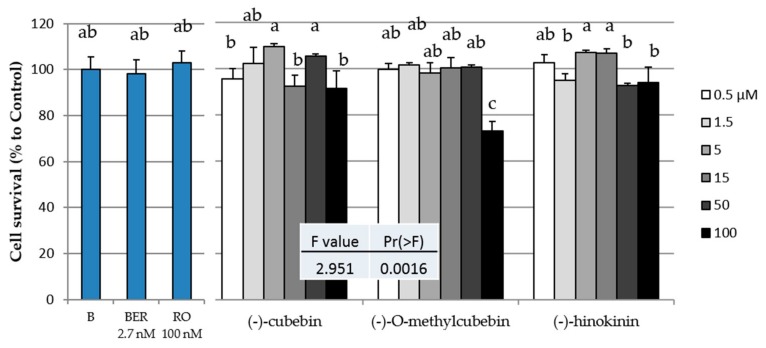
Cytotoxic effects of the three compounds isolated from *V. trifolia*, rosiglitazone (RO), and berberine (BER) in 3T3-L1 cells. B(Black): Cultrued cells without any compund. Data are expressed as the mean ± SD from three independent experiments. The same letters indicate that there are no differences between those groups, and different letters indicate significant differences (*P* < 0.05).

The toxicities of the three isolated compounds, rosiglitazone (RO), and berberine (BER) in 3T3-L1 cells are shown in Figure 2. (-)-*O*-Methylcubebin (MC) was observed to have significant toxicity at 100 µM. From the results shown in Figure 2, it was considered that all three compounds showed no toxicity at 50 µM or less, and thus, their subsequent biological activities were evaluated at 50 µM or less.

### 2.4. Upregulation of Intracellular Lipid Accumulation 

**Figure 3:**
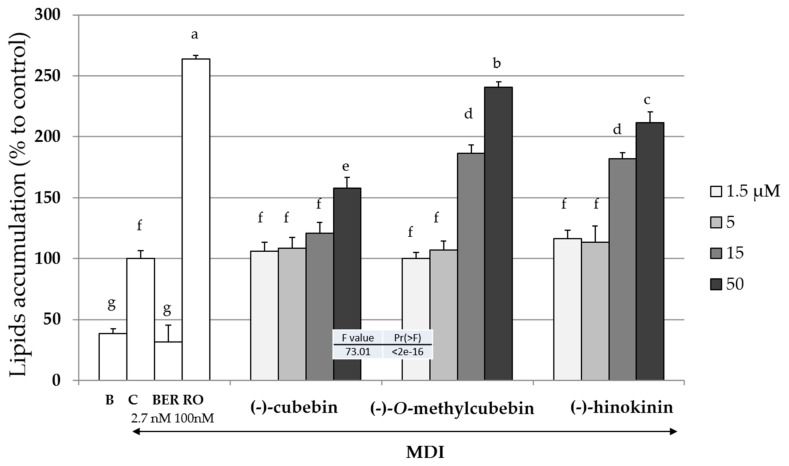
The effects of the compounds isoloated from *V. trifolia* on the liped levels in 3T3-L1 cells. B (Black): Undifferentiated cells without the addition of the MDI mixture [a mixture of 0.5 mM 3-isobuty-1-methyl xanthine (M), 0.1 µM dexamethasone (D) and 2 µM insulin (I)], C (Control): cells with the addition of the MDI, BER: cells with MDI and berberine (2.7 nM), and RO: cells with MDI and rosiglitazone (100 nM). The arrow indicated the addition of the MDI mixture. Data are presented as the mean ± SD from three independent experiments. The same letters indicate that there are no differences between those groups, and different letters indicate significant differences (*P* < 0.05).

The inductions of intracellular lipid accumulation of the three isolated compounds, RO, and BER during the adipogenesis of 3T3-L1 cells with or without MDI (a mixture of 0.5 mM of 3-isobutyl-1-methyl xanthine (M), 0.1 µM of dexamethasone (D), and 2 µM of insulin (I)) mixture are shown in Figure 3. BER or RO decreased or increased intracellular lipids accumulation, respectively. All three isolated compounds increased the intracellular lipids accumulation in a concentration-dependent manner. Among the three compounds, MC had the strongest effect. Thereafter, the effects of only MC were investigated.

### 2.5. Effect of MC on 3T3-L1 Cell Size after Adipogenesis

**Figure 4:**
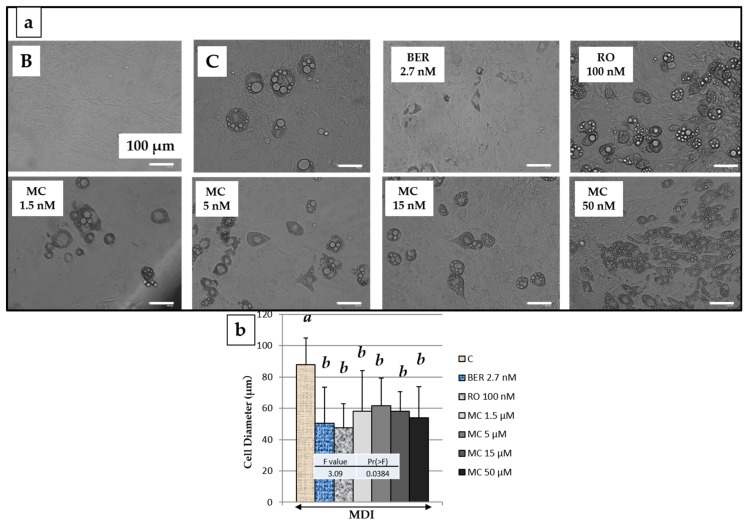
Images and the diameter of 3T3-L1 cells on the say 8 with reference compounds or methylcubelin (MC) of various concentrations. (**a**) Images of 3T3-L1 cells treated with corresponding conditions; (**b**) Cell diameters were determined using Image J software. B (Black): Undifferentiated cells without the addition of the MDI mixture. C (Control): cells with the addition of the MDI Arrow (solid line) indicates the addition of the MDI mixture. Data are presented as the mean ± SD from 100 cells of three independent pictures. The same letters indicate that there are no differences between those groups, and different letters indicate significant differences (*P* < 0.05).

Next, the effect of MC on the differentiated adipocyte size was measured, and the results are shown in Figure 4. It was confirmed that the size of differentiated 3T3-L1 cells was significantly reduced by the addition of rosiglitazone (RO) (100 nM), berberine (BER) (2.7 nM), and MC to the culture medium (Figure 4b). On the other hand, although addition of BER induced downregulation of TG accumulation, size of differentiated cells was almost equal to RO. Thus, it was considered that BER decreased the number of differentiated cells, while reducing the cell diameter.

### 2.6. Effect of MC on Expression of Adipogenesis-Related Proteins, Adiponectin, and GLUT4 in Membrane

**Figure 5:**
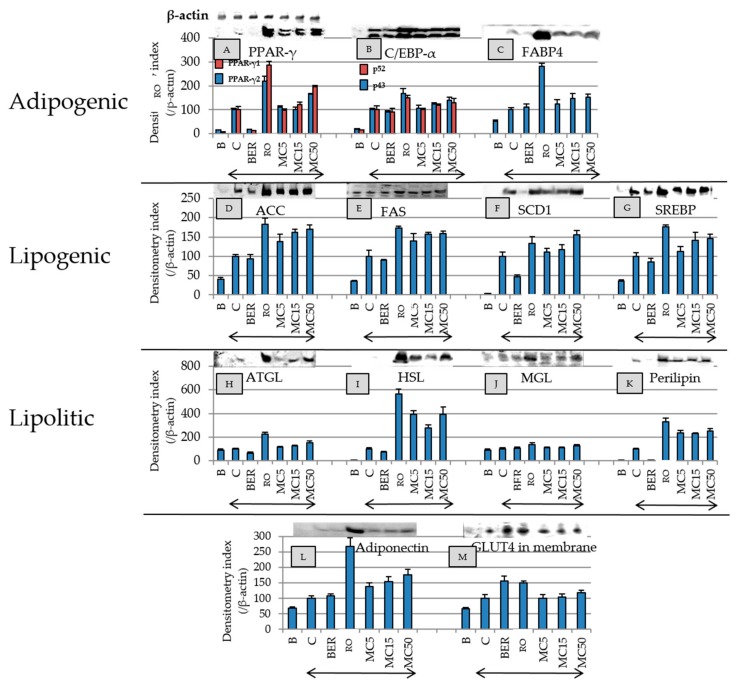
The effects of each compound on adipogenesis-related proteins, adiponectin, and GLUT4 levels in 3T3-L1 cells on day 8. Since all the same samples were used and meansurements were performed using 13 types of primary antibodies, only one β-actin image was shown. Protein levels were measured by electroblotting. B (Black): Undifferentiated cells without the addition of the MDI, C (Control): cells with the addition of the MDI, BER: cells with MDI and berberine (2.7 nM), and RO: cells with MDI and rosiglitazone (100 nM). The number with MC indicate the concentration (µM). Arrows indicate the addition of the MDI mixture. Data are presented as the mean ± SD from three independent experiments. The test results of the significance of protein expression levels are summarized in Table S2 of the Supplemetary Materials.

The effect of MC on adipogenesis-related protein expression was compared to those of reference compounds (RO and BER) (Figure 5). RO increased the expression levels of all of the proteins shown in Figure 5, and MC, such as RO, concentration-dependently increased the concentrations of the all proteins except HSL. MC and RO also increased the expression of adiponectin. RO increased the amount of GLUT4 in the cell membranes, but the addition of MC was not increased by it. BER downregulated the expressions of PPARγ, SCD1, and perilipin, but the expression level of adiponectin was not affected by BER. 

### 2.7. Effects of MC on Intracellular-Signal-Transduction-Related Kinases

**Figure 6:**
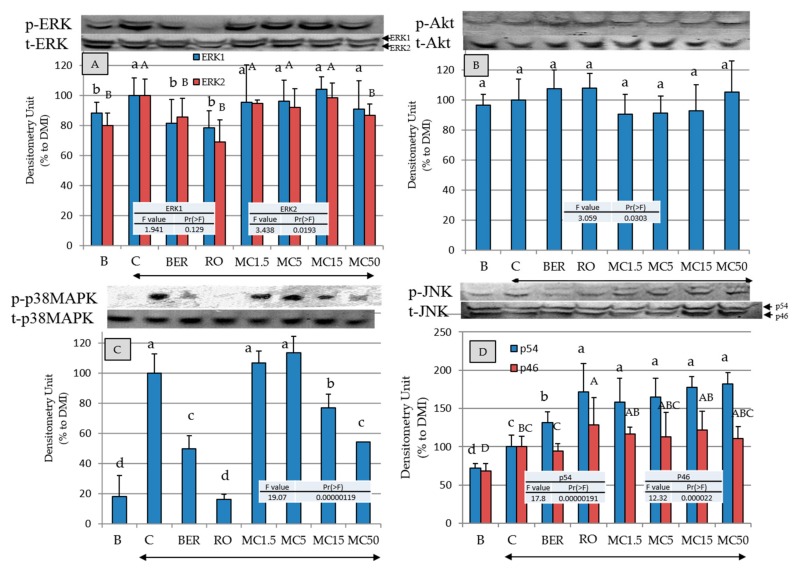
The effects of each compound on the intracellular signal transduction-related protein levels in 3T3-L1 cells on day 8. Protein levels were measured by electroblotting. B (Black): Undifferentiated cells without the addition of the MDI, C (Control): cells with the addition of the MDI, BER: cells with MDI and berberine (2.7 nM), and RO: cells with MDI and rosiglitazone (100 nM). The number with MC indicate the concentration (µM). Arrows indicate the addition of the MDI mixture. Data are presented as the mean ± SD from three independent experiments. The same letters indicate that there are no differences between those groups, and different letters indicate significant differences (*P* < 0.05).

To clarify the mechanisms of the promotion of adipogenesis by MC, the effects on the phosphorylation of the kinases (REK1/2, Akt, p38MAPK, and JNK) in intracellular signal transduction were evaluated (Figure 6). MC, RO, and BER significantly suppress ERK1/2 (only ERK2 at MC50 µM is statistically significant) and p38MAPK phosphorylation, and the phosphorylation of p54JNK and Akt was significantly or statistically not significantly promoted, respectively.

### 2.8. Effects of Specific Kinase Inhibitors and PPAR-γ Antagonist on Promotion of Intracellular Lipid Accumulation by MC 

**Figure 7:**
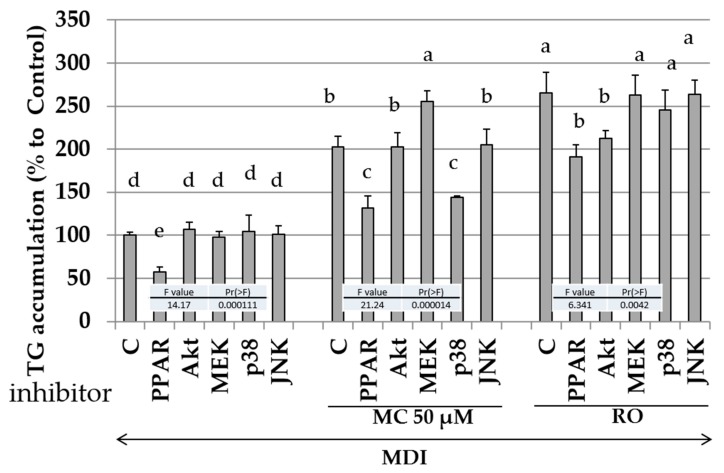
The effects of each inhibitor on the lipid levels in 3T3-L1 cells during adipogenesis. B (Black): Undifferentiated cells without the addition of the MDI, PPAR: cells with MDI and bisphenol A diglycidyl ether, AKt: cells with MDI and wortmannin, MEK: cells with MDI and U0126, p38: cells with MDI and SB202190, and JNK: cells with MDI and JNK inhibitor. The data are presented as the mean ± SD from three independent experiments. The arrow indicates the addition of the MDI mixture. The same letters indicate that there are no differences between those groups, and different letters indicate significant differences (*P* < 0.05).

From the results of Figure 6, MC statistically significantly or not significantly regulated four intracellular signaling transduction-related kinases. Next, the effects of the PPARγ antagonist and specific kinase phosphorylation inhibitor on the promotion of intracellular lipid accumulation by MC were measured (Figure 7). Since inhibitors other than bisphenol A diglycidyl ether (BADGE: an antagonist of PPARγ), i.e., wortmannin (Akt inhibitor), U0126 (inhibitor of MEK1/2 which existing upstream of ERK1/2), SB202190 (p38MAPK inhibitor), and JNK inhibitor did not regulate adipogenesis by MDI solution without MC and RO, effect of each inhibitor on adipogenesis was considered to be negligible at the concentration of 30 µM (left side of Figure 7). Since BADGE suppressed the lipid accumulation effect of the MDI mixture, MC, and RO, MC was considered to be an agonist of PPARγ as well as RO. Compared with the addition of the MDI mixture alone (the leftmost columns in Figure 7), the combined use of the MDI mixture and MC increased the amount of intracellular lipid accumulation (the middle columns in Figure 7). Additionally, the combined use of MDI solution, MC with MEK inhibitor, or p38MAPK inhibitor increased intracellular lipid accumulation statistically significantly. Therefore, it was considered that intracellular lipid accumulation was increased by inhibition of MEK1/2 and p38MAPK. In other words, it was deduced that MC enhanced adipogenesis via inhibition of MEK and p38MAPK. On the other hand, since the effect of RO was suppressed by wortmannin, although, RO only showed the tendency toward upregulation of activation of Akt (Figure 6), RO promoted adipogenesis via the upregulation of the phosphorylation of Akt in the present experimental system.

## 3. Discussion

Three known lignans were isolated from *V. trifolia* in this study. To date, there have been no reports of the isolation of these lignans from plants of the genus *Vitex*. This is the first report on the single-crystal X-ray analysis of (-)-*O*-methylcubebin (MC). Thus far, the affinity of hinokinin for aldose reductase has been estimated to be effective for type 2 diabetes [25]. The effect of (-)-cubebin on 3T3-L1 cells has been reported by Muhammad et al. [26]. Antibacterial activity [27] and analgesic activity [28] by (-)-*O*-methylcubebin have been reported, but we could not find any reports concerned with the antidiabetic or anti-obesity effects of MC.

The compounds treated in this study were isolated from food, and it was assumed that the biological activity is less effective than pharmaceuticals. Therefore, the experimental system used in this study is a model that prevents obesity or diabetes by taking compounds prophylactically in a healthy state. Thus, the test compounds were added to the medium before initiating adipocyte differentiation.

The strength turn of the upregulation of the intracellular lipid accumulation among the three compounds isolated in this study is MC > hinokinin > (-)-cubebin, respectively (Figure 3). Since the polarities of the three compounds are MC < hinokinin < (-)-cubebin, it was considered that the polarity of the compounds and the biological activity were inversely correlated. 

When hypertrophic adipocytes are moved away from the bloodstream, the energy supply tends to be interrupted, and inflammation is induced to worsen diabetes [29]. On the other hand, Meyer et al. reported that the adipose cell size and the amount of adiponectin expression are inversely correlated [30]. From Figure 4, RO and BER, which were used as controls, and MC significantly reduced the diameter of differentiated adipocytes. Additionally, from Figure 5, RO and MC increased the expression of adiponectin, which has the effect of reducing insulin resistance, but BER did not enhance its expression. Therefore, it was found that BER does not increase the expression of adiponectin despite decreasing the differentiated adipocyte diameter.

RO increases the concentration of the adipogenesis-related proteins listed in Figure 5 and acts as an agonist of PPARγ, thus improving diabetes by reducing insulin resistance [31]. Since MC showed the same action as RO but did not change the amount of GLUT4 in the cell membrane compared to CTRL, glucose uptake by GLUT4 may be reduced compared to using RO, and, as a result, MC may have a RO-like action without weight gain. Thus, MC may have RO-like effects without the side effects of weight gain. Therefore, MC may be a candidate lead compound for antidiabetic drugs that compensates for the weaknesses of RO.

BER is an alkaloid compound having bitterness which is contained in the *Coptis japonica* and *Phellodendron amurense* and shows an improvement effect on type 2 diabetes. Moreover, BER improved blood sugar levels in the patients with type 2 diabetes, and the effect may be similar levels of RO [32]. BER also suppresses lipid accumulation in adipocyte. Thus, RO improves diabetes through the induction of adipogenesis and intracellular lipid accumulation, while BER may suppress intracellular lipid accumulation and improve diabetes. In this study, we used RO and BER as antidiabetic compounds with different mechanisms. In Figure 5A–D,F–I,K, MC clearly shows a similar effect of RO, and MC may act to improve diabetes with a similar mechanism to RO. 

On the other hand, the addition of BER reduced the expression levels of PPARγ, SCD1, and perilipin (Figure 5). PPARγ is a nuclear receptor that triggers adipocyte differentiation [33], and perilipin is expressed specifically in adipocytes and lipid droplets surrounded by perilipin that are known to be less susceptible to hydrolysis by lipolytic enzymes [34]. Since BER significantly suppressed the expression of PPARγ and perilipin compared to the control, it is considered that BER suppressed intracellular fat accumulation by inhibition of the initiation of adipogenesis and the promotion of lipid droplet degradation.

A previous report showed that the BER improves diabetes by an enhanced insulin receptor (IRS) expression [35]. Thus, using by Western blot method, we measured the IRS phosphorylation levels at 30 min and the IRS expression levels at 8 days after the stimulation. As a result, no significant difference between addition of MC and BER was seen (data not shown).

It has also been reported that the effects of BER are unaffected by Akt inhibitors but are acted additively to MEK inhibitors [36]. Therefore, the effects of BER on ERK1/2 and Akt obtained in this study were consistent with the results of previous studies.

It is known that obesity and diabetes are also regulated by intracellular-signal-reduction-related kinases [37]. Figure 6 shows that RO, BER, and MC inhibited ERK1/2 and p38MAPK phosphorylation and promoted JNK phosphorylation. Insulin has been shown to promote the migration of GLUT4 from the cytoplasm to the cell membrane via the phosphorylation of Akt [38]. Figure 6 shows that the addition of RO, BER, and MC tended to promote phosphorylation of Akt, and RO and BER increased the content of GLUT4 in the cell membrane, but MC did not show the effect of increasing the abundance of GLUT4 in the cell membrane (lower right of Figure 5).

To further confirm the relationship between intracellular-signal-transduction-related kinases and MC, the effects of specific kinase inhibitors were examined (Figure 7). Since BADGE, an antagonist of PPARγ, suppressed the intracellular lipid accumulation by MC and RO, MC is considered to act as an agonist of PPARγ such as RO. Moreover, since the effect of MC was enhanced by U0126 and SB202190 and was unrelated to wortmannin and JNK inhibitor, the action of MC was obtained by the downregulation of the phosphorylation of ERK1/2 and p38MAPK but was unrelated to the activation or inactivation of Akt or JNK. Since the action of RO was reduced by wortmannin, it is considered that the action of RO can be obtained through the phosphorylation of Akt in the experimental system of this study.

From the above results, MC regulated the adipogenesis of 3T3-L1 cells by acting as a ligand for PPARγ and downregulating the phosphorylation of ERK1/2 and p38MAPK, and it was deduced that adiponectin expression was enhanced with the reduction of the differentiated adipocyte size.

It is known that the inactivation of ERK1/2 enhances the binding ability of PPARγ to DNA [39], and activation induces the phosphorylation of PPARγ, followed by attenuation of the transcriptional activity [40]. On the other hand, it was reported that, similar to ERK1/2, activated JNK induced the phosphorylation of PPARγ [40]. In the present study, the biological activity of MC was not changed by the addition of JNK inhibitor to the medium, so JNK phosphorylation by MC (Figure 6) is considered to be unrelated to the enhancement of intracellular lipid accumulation by MC. Additionally, there are reports that activation of P38MAPK promotes adipogenesis and glucose absorption [41,42]. In the present study, MC promoted adipocyte differentiation via inactivation of p38MAPK. The relationship between MC’s enhancement of intracellular lipid accumulation and p38MAPK needs to be clarified through experiments with animals.

It has already been reported that apoptosis is enhanced by inhibiting ERK1/2 activity with siRNA in Hela cells [43]. By adding MC to a system in which the activity of each MAPK is inhibited by siRNA, the action mechanism of MC is likely to be clarified.

Adiponectin, a circulating protein derived from adipocytes, is a typical adipocytokine and has two main characteristics. (1) Its circulating concentration is about 3 to 6 orders of magnitude higher than normal hormones and cytokines [44]. (2) Its concentration is inversely related to body fat mass, despite adipocyte-specific production. In addition, administration of antidiabetic drugs increases adiponectin levels and decreases insulin resistance and endothelial dysfunction [45]. Obesity also induces adiponectin reduction with increased insulin resistance [46].

Both RO and MC used as positive controls in this study enhanced adipocyte differentiation, increased intracellular lipid accumulation, and increased adiponectin expression. On the other hand, BER, which has an antidiabetic effect similar to RO, decreased lipid accumulation in adipocytes and adiponectin expression. Therefore, the increased expression of adiponectin may not be correlated with antidiabetic properties.

The mRNA of adiponectin, which is one of the key compounds in the treatment of diabetes, is expressed by the binding of retinoid X receptor (RXR) and activated PPARγ to peroxisome proliferator response element (PPRE) and Liver Receptor Homolog-1 (LRH-1) binding to LRH-RE on DNA [47]. Although this study only revealed that MC is an agonist of PPARγ, we believe that evaluation of the effects of MC on RXR and LRH-1 may reveal the full effect of MC on the transcription factor of adiponectin [48].

BER reportedly enhances the expression of adiponectin through the activation of AMPK [49]. From Figure 5L, no increase of adiponectin was observed in 3T3-L1 cells stimulated with BER. To clarify the relationships between BER and adiponectin expression in the 3T3-L1 cells, additional studies regarding detailed adiponectin levels at various BER concentrations may be needed.

Brown and beige adipocytes are considered to have the ability to consume chemical energy and generate heat for preventing obesity and metabolic disorders [50]. Recently, it has been reported that capsaicin and capsiate, components of chili, differentiate white adipocytes into brown or beige [51]. The next step in this study is to determine the biological activities of MC that could not be discussed in this study, by measuring the expression of UCP1 and PGC1a via stimulation of preadipocytes.

In the present study, we examined the effects of MC on adipogenesis in vitro. To clarify the possibility that MC will be used as a drug lead compound, it is necessary to confirm its antidiabetic effects similar to that of rosiglitazone, but with less side effects, using experimental animals.

## 4. Materials and Methods 

### 4.1. General Experimental Procedures

A JEOL ECX 400 or 500 spectrometer with tetramethylsilane as an internal standard was used for recording the ^1^H (400 or 500 MHz) and ^13^C (100 or 125 MHz) NMR spectra. A QP-2010 (Shimadzu; Kyoto, Japan) was used for the measurement of electron ionization mass spectrometry (EI-MS). A suitable crystal of the isolated compounds was selected and mounted on a diffractometer (VariMax with Pilatus detector, Rigaku; Tokyo, Japan). The crystal was kept at 293(2) K during data collection. Using Olex2 [52], the structure was solved with the ShelXT [53] structural solution program, using Intrinsic Phasing, and refined with the ShelXL refinement package, using least squares minimization. The validity of the measurement results was confirmed by checkCIF (https://checkcif.iucr.org/). 

The extract was separated by medium pressure liquid chromatography (MPLC) (Yamazen Co., Ltd., Tokyo, Japan), using a silica gel column [Universal column 5 L (φ 64 × 240 mm)], octadecyl silane (ODS) column [Universal column S (φ 18 × 114 mm)], and HPLC with silica gel column (Develosil 60: φ 10 × 250 mm; Nomura Chemical, Aichi, Japan). 

### 4.2. Reagents

All of the organic solvents (n-hexane, ethyl acetate, and methanol) were obtained from Wako Pure Chemical Industries (Osaka, Japan). The 3-Isobutyl-1-methyl xanthine (M), dexamethasone (D), insulin (I), and Dulbecco’s Modified Eagle’s Medium (DMEM) were purchased from Sigma-Aldrich (St. Louis, MO, USA). Fetal bovine serum (FBS) and calf serum (CS) from Gibco BRL (Gaithersburg, MD, USA) were used with DMEM. A peroxisome proliferator-activated receptor γ (PPARγ) inhibitor (bisphenol A diglycidyl ether; BADGE), a phosphoinositide 3-kinase (PI3K)/Akt inhibitor (wortmannin), a mitogen-activated protein kinase (MAPK)/extracellular signal-regulated kinase (ERK) kinase (MEK) inhibitor (U0126), and a p38MAPK inhibitor (SB202190) were obtained from TCI (Tokyo, Japan). JNK inhibitor (CAS 129-56-6; Figure S2 in the Supplementary Materials) was purchased from Merck (Darmstadt, Germany).

### 4.3. Plant Material

The leaves of *Vitex trifolia* L. were purchased commercially in Yangon, Myanmar, and were identified by Dr. Nyunt Phay (Rector, Pathein University, Pathein, Myanmar). The voucher specimen has been deposited as MY-33 in the College of Science and Technology, Nihon University, Tokyo, Japan.

### 4.4. Preparation of Solvent Extracts

Powdered leaves of *Vitex trifolia* L. (20 kg) were immersed in 100 L of methanol and extracted at room temperature for 24 h. Subsequently, methanol containing the extracts was filtered through filter paper (No. 2; Advantech, Tokyo, Japan), and the filtrate was evaporated by a vacuum dryer (Iwai Co., Ltd., Shizuoka, Japan) to obtain 2.4 kg of the crude methanol extract. The crude methanol extract was stored at −20 °C until use. 

The crude methanol extract (200 g) was immersed in *n*-hexane (1 L) for 24 h, at room temperature. The solvent containing the extracts was filtered through filter paper (No. 2; Advantech), and the filtrate was dried in vacuo to prepare the hexane extract. The residue was then immersed in ethyl acetate (1 L) at room temperature for 24 h and filtered, and the filtrate was dried in vacuo, to prepare the ethyl acetate extract. Next, the methanol extract was obtained in the same manner.

### 4.5. Isolation of Compounds

The obtained ethyl acetate extract (10.0 g) was fractionated by silica gel medium pressure column chromatography (MPLC) (*n*-hexane: ethyl acetate = 100:0 to 0:100) to obtain 16 fractions (fractions 1–16). Fractions 6 (30.4 mg) and 7 (28.8 mg), in which single spots were detected by thin-layer chromatography (TLC) analysis, were purified by octa decyl silyl (ODS) column MPLC (methanol:water, 0:100 to 100:0), followed by high-pressure liquid chromatography (HPLC) with silica gel column purification. Compounds **1** [(-)-Cubebin] (3.9 mg) and **2** [(-)-*O*-Methylcubebin] (4.8 mg) were isolated, respectively. On the other hand, fraction 12 (19.1 mg) was fractionated by ODS MPLC (methanol:water, 0:100 to 100:0), to obtain fractions (fractions 17–25). Fraction 21 was fractionated by ODS MPLC (methanol:water, 0:100 to 100:0), to obtain 4 fractions (fractions 26–29). Fraction 27, in which a single spot was observed, was purified by HPLC, and 3.2 mg of compound **3** [(-)-Hinokinin] was isolated (see Supplementary Materials, Figure S3 and Table S1).

(-)-Cubebin (**1**): Yellow crystal; ^1^H NMR (chloroform-*d*, 400 MHz) δ 6.68–6.63 (2H, m), 6.61 (2H, d, *J* = 7.9 Hz), 6.57–6.51 (1H, m), 6.50–6.46 (2H, m), 6.46–6.42 (2H, m), 5.86 (3H, s), 5.85 (3H, s), 5.85 (2H, d, *J* = 1.2 Hz), 5.15 (2H, d, *J* = 1.8 Hz), 4.08–3.85 (1H, m), 3.84–3.64 (1H, m), 2.63–2.54 (1H, m), 2.54–2.41 (2H, m), 2.41–2.29 (1H, m), 2.08 (2H, tdd, *J* = 7.7, 6.1, 3.9 Hz); ^13^C NMR (chloroform-*d*, 100 MHz) δ 147.8, 147.7, 146.0, 145.9, 134.2, 133.4, 121.9, 121.5, 109.4, 109.3, 108.3, 108.3, 103.5, 101.97, 100.92, 72.4, 52.2, 46.0, 39.0, 38.5: EI-MS; *m/z* [M]^+^ 356; Crystal data for C_20_H_20_O_6_ (*M* =356.36 g/mol): monoclinic, space group P2_1_ (no. 4), *a* = 11.60980(10) Å, *b* = 5.51720(10) Å, *c* = 13.40780(10) Å, *β* = 100.0820(10)°, *V* = 845.556(18) Å^3^, *Z* = 2, *T* = 293(2) K, µ(CuKα) = 0.860 mm^−1^, *Dcalc* = 1.400 g/cm^3^, 12,275 reflections measured (6.696° ≤ 2Θ ≤ 147.934°), 3204 unique (*R*_int_ = 0.0340, R_sigma_ = 0.0185), which were used in all calculations. The final *R*_1_ was 0.0321 (*I* > 2σ(*I*)), and *wR*_2_ was 0.1038 (all data). The goodness of fit on F^2^ was 0.990, and the Flack parameter = 0.02(16).

(-)-*O*-Methylcubebin (**2**): Clear crystal; ^1^H NMR (methanol-*d*_4_, 500 MHz) δ 6.75–6.63 (5H, m), 6.66–6.59 (4H, m), 5.92–5.85 (6H, m), 4.61 (1H, s), 3.92 (1H, t, *J* = 8.4 Hz), 3.57 (1H, dd, *J* = 8.5, 7.0 Hz), 3.28 (4H, s), 2.66 (3H, ddd, *J* = 13.5, 7.8, 4.0 Hz), 2.56–2.40 (3H, m), 2.32 (2H, qt, *J* = 8.8, 6.1 Hz), 1.99 (1H, tt, *J* = 9.9, 5.1 Hz); ^13^C NMR (methanol-*d*_4_, 125 MHz) δ 147.9, 147.7, 146.1, 145.9, 134.8, 134.1, 121.4, 121.3, 108.9, 108.7, 107.7, 107.7, 100.8, 100.7, 71.8, 53.5, 52.0, 43.4, 38.9, 33.3; EI-MS; *m/z* [M]^+^ 370.; Crystal data for C_22_H_23_O_5_ (M =367.40 g/mol): monoclinic, space group P2_1_ (no. 4), *a* = 10.6931(17) Å, *b* = 5.8843(7) Å, *c* = 15.058(3) Å, *β* = 108.131(17)°, *V* = 900.4(2) Å^3^, *Z* = 2, *T* = 293(2) K, µ(CuKα) = 0.780 mm^−1^, *Dcalc* = 1.355 g/cm^3^, 14,097 reflections measured (6.176° ≤ 2Θ ≤ 150.172°), 3572 unique (*R_int_* = 0.0800, *R_sigma_* = 0.0789), which were used in all calculations. The final *R_1_* was 0.0987 (*I* > 2σ(*I*)), and *wR_2_* was 0.2711 (all data). The goodness of fit on *F^2^* was 1.252, and the Flack parameter = 0.0(2).

(-)-Hinokinin (**3**):Yellow gum; ^1^H NMR (chloroform-*d*, 500 MHz) δ 6.79 (1H, s), 6.77 (1H, dd, *J* = 8.4, 5.0 Hz), 6.73–6.64 (2H, m), 6.62 (1H, d, *J* = 1.8 Hz), 6.60–6.46 (2H, m), 6.04–5.81 (2H, m), 4.25–4.00 (1H, m), 3.86 (1H, s), 2.94 (1H, dd, *J* = 11.3, 6.0 Hz), 2.85–2.68 (1H, m), 2.66–2.53 (2H, m), 2.45–2.36 (1H, m); ^13^C NMR (chloroform-*d*, 125 MHz) δ 178.5, 147.9, 147.9, 146.5, 146.4, 131.6, 131.3, 122.2, 121.6, 109.5, 108.8, 108.4, 108.3, 101.0, 71.2, 46.5, 38.4, 34.9; EI-MS; *m/z* [M]^+^ 354.

### 4.6. Cell Culture 

The 3T3-L1 cells, which were purchased from Riken Cell Bank (Tsukuba, Japan), were passaged 3 times in Dulbecco’s modified eagle medium (DMEM) (high glucose content) with 10% calf serum. Cells in DMEM supplemented with 10% CS were seeded at 5 × 10^3^ cells/well onto 24-well plates (Corning Life Sciences, Lowell, MA, USA) for 2 or 3 days, until 80% confluence (day 0), and the medium was replaced with DMEM containing 10% FBS and a mixture of 0.5 mM 3-isobutyl-1-methyl xanthine (M), 0.1 µM dexamethasone (D), and 2 µM insulin (I) (MDI mixture), with or without one of the test compounds, rosiglitazone (RO) (0.1 µM), or berberine (BER) (2.7 nM). After 48 h (day 2), the medium was replaced with DMEM containing 10% FBS and 2 µM of insulin. After 48 h (day 4), the medium was replaced with DMEM containing 10% FBS. Thereafter, the medium was exchanged every other day. Cells were maintained in a humidified atmosphere of 5% CO_2_ at 37 °C. All test compounds were dissolved in DMSO, filter-sterilized, and stored at −20 °C until just before use (see Supplementary Materials, Figure S4)

### 4.7. Cell Toxicity Assay

The 3T3-L1 cells were seeded at 5 × 10^3^ cells/well on a 96-well plate and cultured in DMEM with 10% calf serum, until 80% confluence. The medium was replaced with DMEM containing 10% FBS with or without test compound. A Cell Counting Kit-8 (Dojindo, Kumamoto, Japan) was used for the measurement of the cell viability after 48 h of culture, according to the manufacturer’s instructions.

### 4.8. Measurement of the Intracellular Lipid Level

The 3T3-L1 cells were cultured in 24-well plates and cultured in DMEM with 10% calf serum until 80% confluence. The cells were differentiated with a mixture of 0.5 mM of 3-isobutyl-1-methyl xanthine (M), 0.1 µM of dexamethasone (D), and of 2 µM insulin (I) (MDI mixture) and each compound. The intracellular lipid levels in 3T3-L1 cells at day 8 were measured by an E-test WAKO Triglyceride Kit (Wako Pure Chemical), according to the manufacturer’s instructions.

### 4.9. Measurement of Adipose Cell Size

Live images of 3T3-L1cells were taken by a fluorescent cell imager (Floid Cell Imaging Solution; Thermo Fisher Scientific, Waltham, MA, USA). The average size of adipose cells at day 8 was measured by Image J software (version 1.52, Research Services Branch, National Institute of Mental Health, Bethesda, MD, USA) [54]. 

### 4.10. Protein Detection 

Differentiated (day 8) 3T3-L1 cells in 6-well plates were placed on ice, and each well was washed with PBS and subsequently lysed with 150 µL of 20 mM Tris-HCl buffer (pH = 8.0) containing 150 mM of NaCl, 2 mM of EDTA, 1% Nonidet P-40 (*w/v*), 1% sodium deoxycholate (*w/v*), 0.1% sodium dodecyl sulfate (*w/v*), 50 mM of NaF, 0.1% aprotinin (*w/v*), 0.1% leupeptin (*w/v*), 1 mM of Na_3_VO_4_, and 1 mM of phenylmethylsulfonylfluoride (PMSF). Cell lysates were collected by using a cell scraper and were centrifuged at 15,000× *g* for 30 min at 4 °C. The supernatant was collected, and the overall protein concentration was determined using a Protein Assay Reagent Kit (Cytoskeleton, Denver, CO) with BSA as the standard. To detect GLUT4, membrane protein was extracted using a Plasma Membrane Protein Extraction Kit (101 Bio, Palo Alto, CA, USA) according to the instructions of the manufacturer.

Supernatant fluids containing proteins were mixed with lithium dodecyl sulfate (LDS) sample buffer (Invitrogen Corp, Carlsbad, CA, USA) and incubated for 5 min at 80 °C. Samples containing proteins (20 µg) were loaded in each lane, followed by separation by SDS-polyacrylamide gel electrophoresis, and the proteins in gels were electroblotted onto polyvinylidene fluoride (PVDF) filters (Hybond-P, 0.2 µM; GE Healthcare, Little Chalfont, UK). The list of primary and secondary antibodies is summarized in Table S3 of the Supplementary Materials. Before use, primary and secondary antibodies were diluted 1000 or 3000 times, respectively. The enhanced chemiluminescence method (Western Lightning ECL Pro; Perkin Elmer, Waltham, MA, USA) was used for development of the blots [55].

### 4.11. Treatment with Specific Inhibitor

The 3T3-L1 cells were cultured until 80% confluence (day 0), and BADGE, U0126, SB202190, wortmannin, or JNK inhibitor was added to the medium at a final concentration of 100 µM (BADGE) or 30 µM (U0126, SB202190, wortmannin, and JNK inhibitor), 1 h before the addition of the MDI mixture, with or without each isolated compound. 

### 4.12. Statistical Analysis 

The results were expressed as the mean ± standard deviation (SD). The significant difference between the groups compared was determined by using one-way analysis of variance (one way-ANOVA).

## Supplementary Materials

The following are available online. Figure S1. Cytotoxic effects and effects for intracellular lipid accumulations of the three extracts from V. trifolia in 3T3-L1 cells. Figure S2. Chemical structure of JNK-inhibitor. Figure S3. Fractionation of ethyl acetate extract. Table S1. Conditions of the chromatography. Figure S4. Cell culture. Table S2. Test result of significance of protein expression level in Figure 5. Table S3. List of used antibodies. Table S4. Chemical shift of the isolated compounds in 13C-NMR Spectra.
