# Supplementary material for: (-)-O-Methylcubebin from Vitex trifolia Enhanced Adipogenesis in 3T3-L1 Cells via the Inhibition of ERK1/2 and p38MAPK Phosphorylation
Source: Molecules. 2019 Dec 24;25(1):73. doi: 10.3390/molecules25010073 (PMC6994966; doi:10.3390/molecules25010073)
Supplement: Supplementary file 1 [file molecules-25-00073-s001.pptx]

## Slide 1
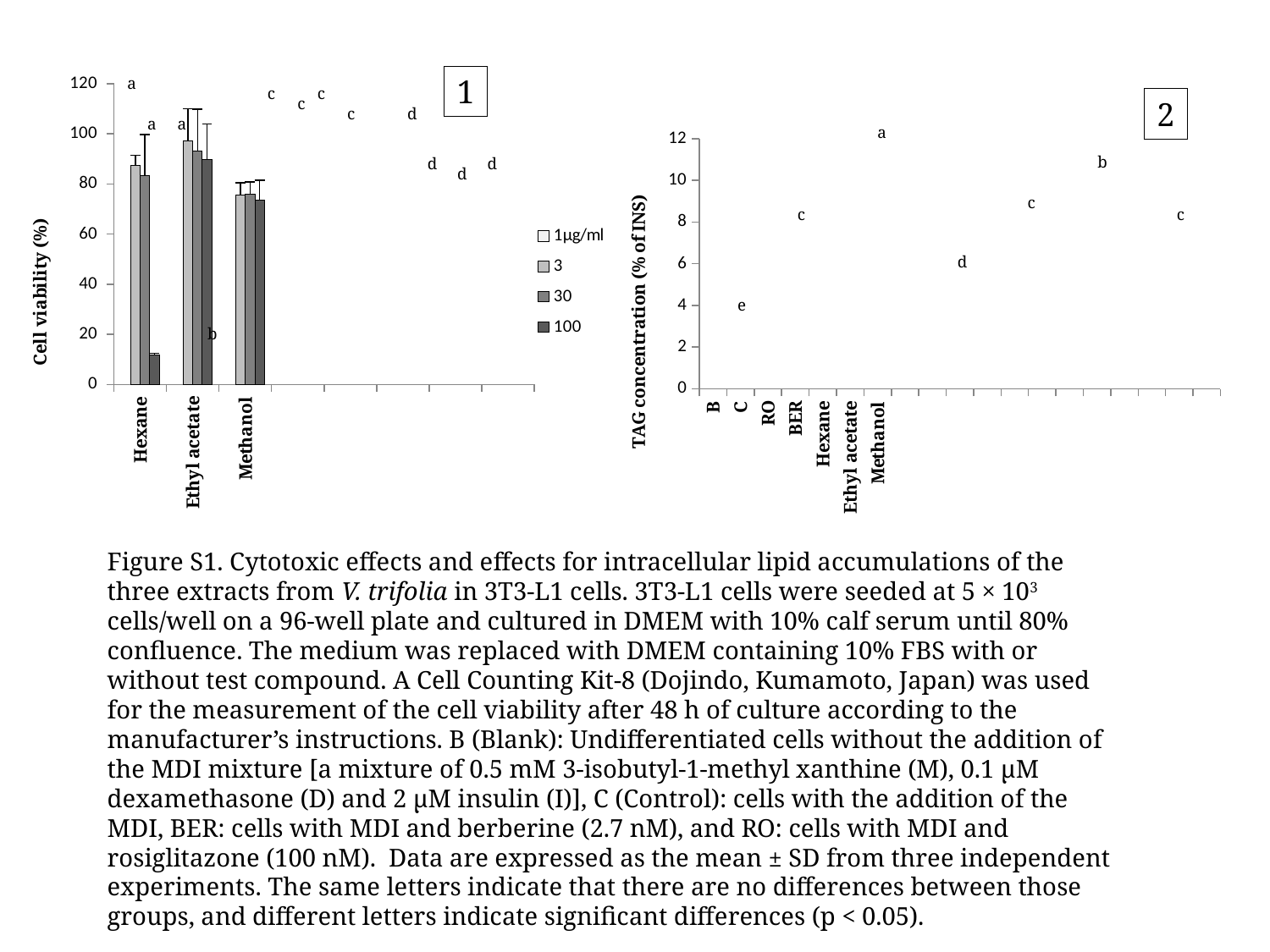

### Chart
| Category | 1μg/ml | 3 | 30 | 100 |
|---|---|---|---|---|
| Hexane | 98.8937093275488 | 87.258 | 83.49240780911063 | 11.778741865509762 |
| Ethyl acetate | 100.02169197396964 | 97.307 | 93.26619150914163 | 89.86365044933376 |
| Methanol | 86.22559652928416 | 75.59652928416486 | 75.79485590331579 | 73.42733188720173 |1
2
### Chart
| Category | |
|---|---|
| B | 37.920937042459734 |
| C | 100.0 |
| RO | 156.1706 |
| BER | 67.5695 |
| Hexane | 105.0 |
| Ethyl acetate | 131.0 |
| Methanol | 103.0 |a
b
c
c
c
d
e
Figure S1. Cytotoxic effects and effects for intracellular lipid accumulations of the three extracts from V. trifolia in 3T3-L1 cells. 3T3-L1 cells were seeded at 5 × 10^3^ cells/well on a 96-well plate and cultured in DMEM with 10% calf serum until 80% confluence. The medium was replaced with DMEM containing 10% FBS with or without test compound. A Cell Counting Kit-8 (Dojindo, Kumamoto, Japan) was used for the measurement of the cell viability after 48 h of culture according to the manufacturer’s instructions. B (Blank): Undifferentiated cells without the addition of the MDI mixture [a mixture of 0.5 mM 3-isobutyl-1-methyl xanthine (M), 0.1 μM dexamethasone (D) and 2 μM insulin (I)], C (Control): cells with the addition of the MDI, BER: cells with MDI and berberine (2.7 nM), and RO: cells with MDI and rosiglitazone (100 nM). Data are expressed as the mean ± SD from three independent experiments. The same letters indicate that there are no differences between those groups, and different letters indicate significant differences (p < 0.05).

## Slide 2
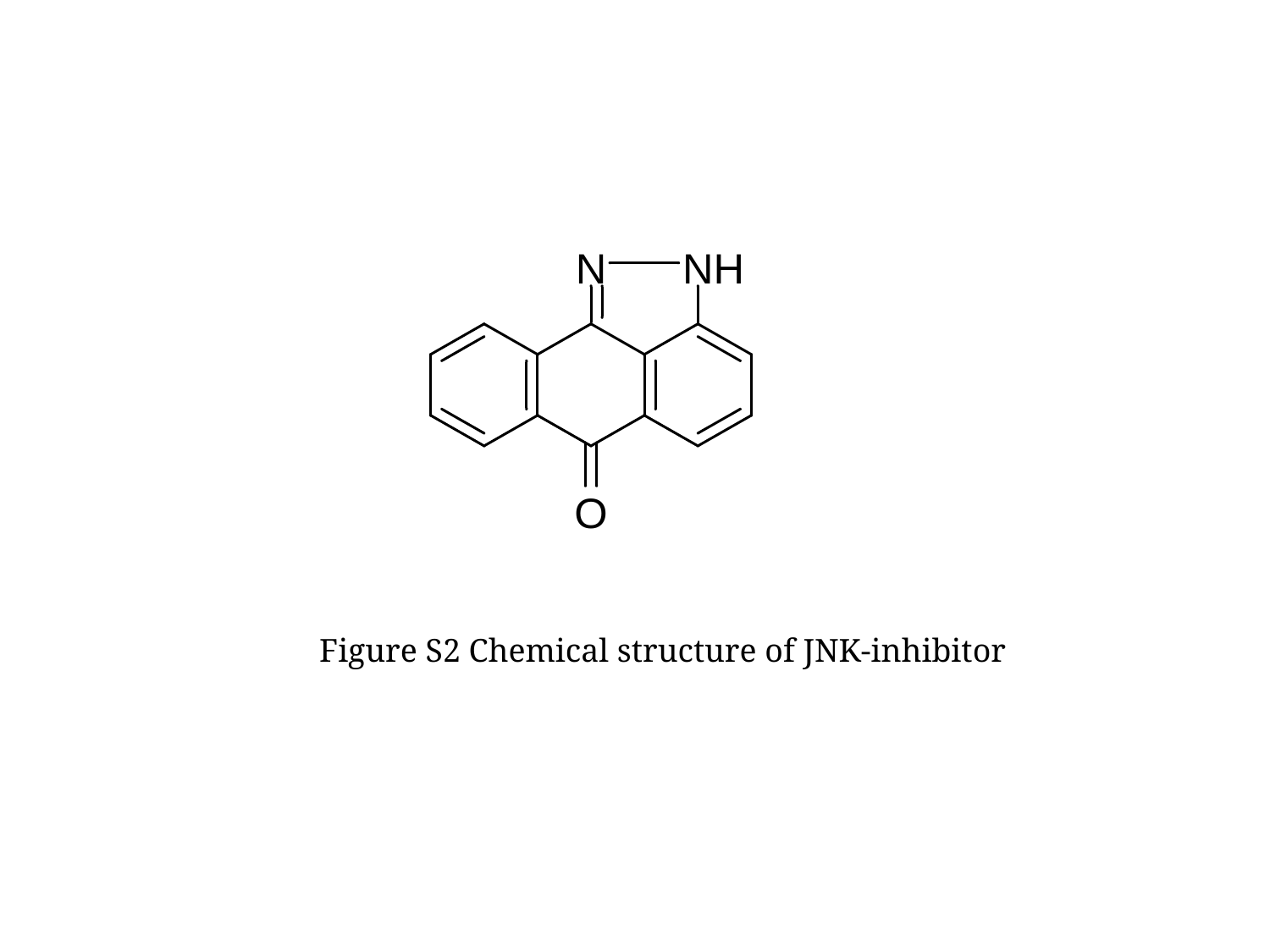

Figure S2 Chemical structure of JNK-inhibitor

## Slide 3
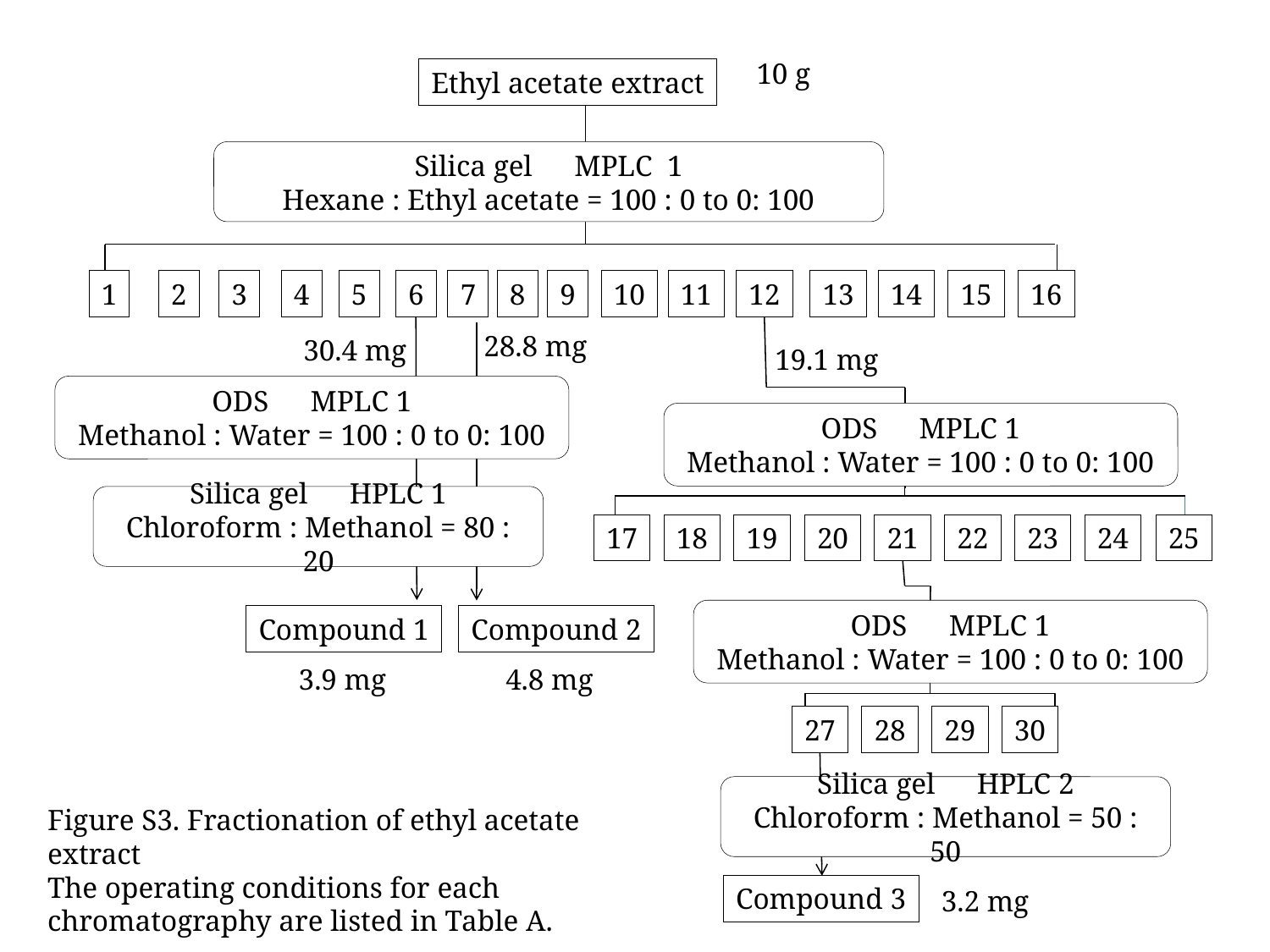

10 g
Ethyl acetate extract
Silica gel　MPLC 1
Hexane : Ethyl acetate = 100 : 0 to 0: 100
1
2
3
4
5
6
7
8
9
10
11
12
13
14
15
16
28.8 mg
30.4 mg
19.1 mg
ODS　MPLC 1
Methanol : Water = 100 : 0 to 0: 100
ODS　MPLC 1
Methanol : Water = 100 : 0 to 0: 100
Silica gel　HPLC 1
Chloroform : Methanol = 80 : 20
17
18
19
20
21
22
23
24
25
ODS　MPLC 1
Methanol : Water = 100 : 0 to 0: 100
Compound 1
Compound 2
3.9 mg
4.8 mg
27
28
29
30
Silica gel　HPLC 2
Chloroform : Methanol = 50 : 50
Compound 3
3.2 mg
Figure S3. Fractionation of ethyl acetate extract
The operating conditions for each chromatography are listed in Table A.

## Slide 4
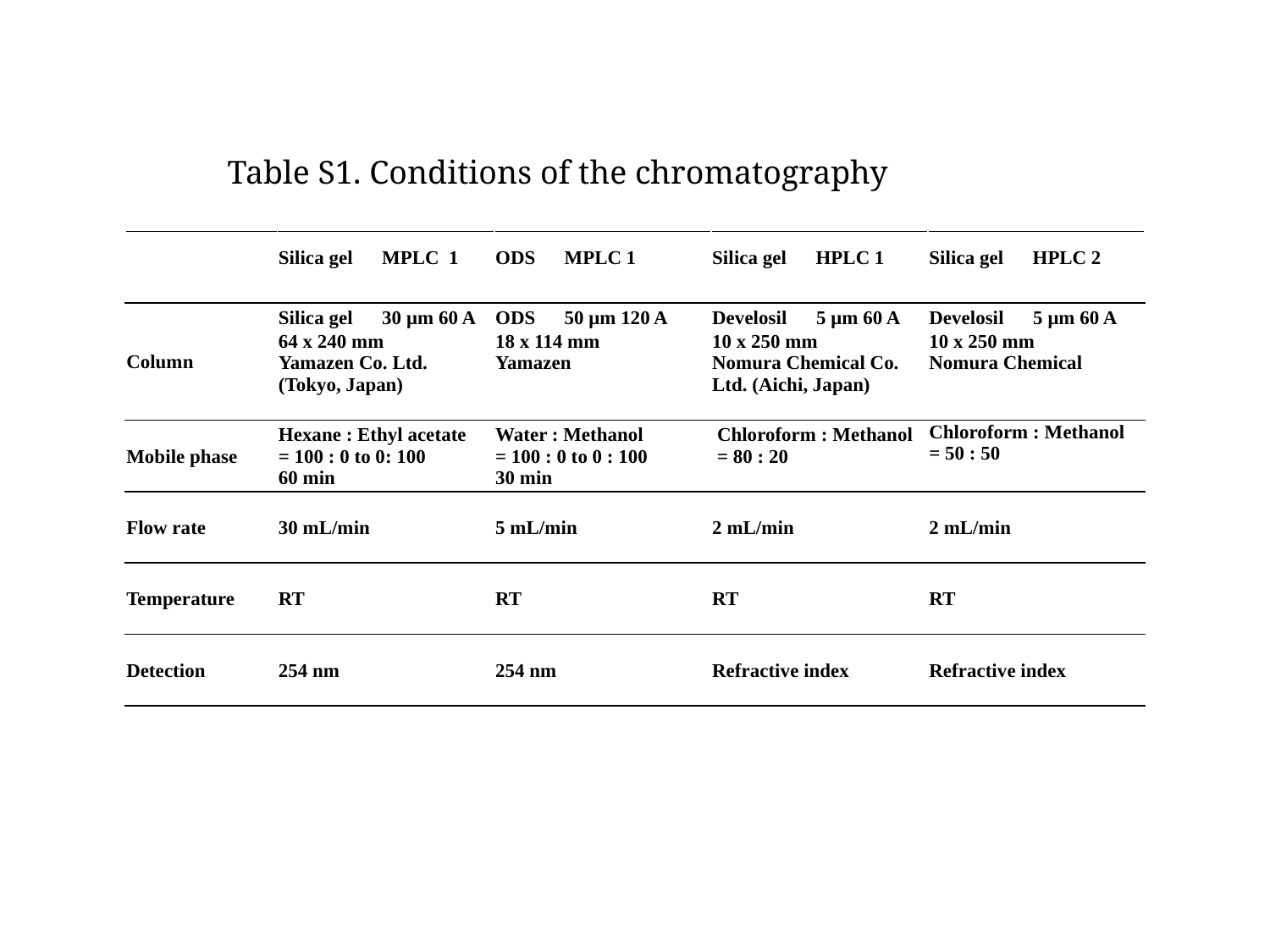

Table S1. Conditions of the chromatography
| | Silica gel　MPLC 1 | ODS　MPLC 1 | Silica gel　HPLC 1 | Silica gel　HPLC 2 |
| --- | --- | --- | --- | --- |
| Column | Silica gel　30 μm 60 A 64 x 240 mm Yamazen Co. Ltd. (Tokyo, Japan) | ODS　50 μm 120 A 18 x 114 mm Yamazen | Develosil　5 μm 60 A 10 x 250 mm Nomura Chemical Co. Ltd. (Aichi, Japan) | Develosil　5 μm 60 A 10 x 250 mm Nomura Chemical |
| Mobile phase | Hexane : Ethyl acetate = 100 : 0 to 0: 100 60 min | Water : Methanol = 100 : 0 to 0 : 100 30 min | Chloroform : Methanol = 80 : 20 | Chloroform : Methanol = 50 : 50 |
| Flow rate | 30 mL/min | 5 mL/min | 2 mL/min | 2 mL/min |
| Temperature | RT | RT | RT | RT |
| Detection | 254 nm | 254 nm | Refractive index | Refractive index |

## Slide 5
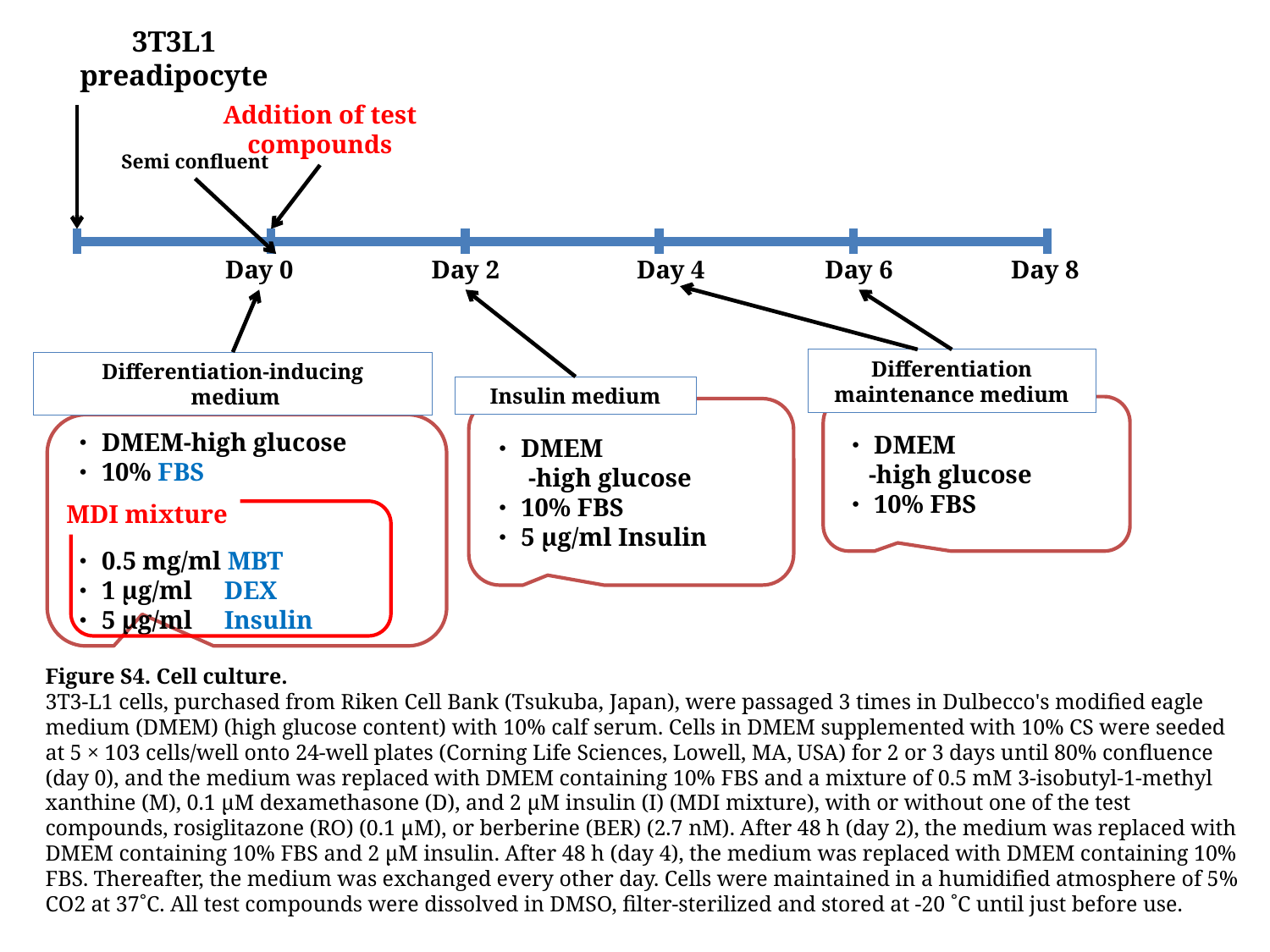

3T3L1 preadipocyte
Addition of test compounds
Semi confluent
Day 0
Day 2
Day 4
Day 8
Day 6
Differentiation maintenance medium
Differentiation-inducing
 medium
Insulin medium
・DMEM
 -high glucose
・10% FBS
・5 μg/ml Insulin
・DMEM
 -high glucose
・10% FBS
・DMEM-high glucose
・10% FBS
・0.5 mg/ml MBT
・1 μg/ml DEX
・5 μg/ml Insulin
MDI mixture
Figure S4. Cell culture.
3T3-L1 cells, purchased from Riken Cell Bank (Tsukuba, Japan), were passaged 3 times in Dulbecco's modified eagle medium (DMEM) (high glucose content) with 10% calf serum. Cells in DMEM supplemented with 10% CS were seeded at 5 × 10^3^ cells/well onto 24-well plates (Corning Life Sciences, Lowell, MA, USA) for 2 or 3 days until 80% confluence (day 0), and the medium was replaced with DMEM containing 10% FBS and a mixture of 0.5 mM 3-isobutyl-1-methyl xanthine (M), 0.1 μM dexamethasone (D), and 2 μM insulin (I) (MDI mixture), with or without one of the test compounds, rosiglitazone (RO) (0.1 μM), or berberine (BER) (2.7 nM). After 48 h (day 2), the medium was replaced with DMEM containing 10% FBS and 2 μM insulin. After 48 h (day 4), the medium was replaced with DMEM containing 10% FBS. Thereafter, the medium was exchanged every other day. Cells were maintained in a humidified atmosphere of 5% CO2 at 37˚C. All test compounds were dissolved in DMSO, filter-sterilized and stored at -20 ˚C until just before use.

## Slide 6
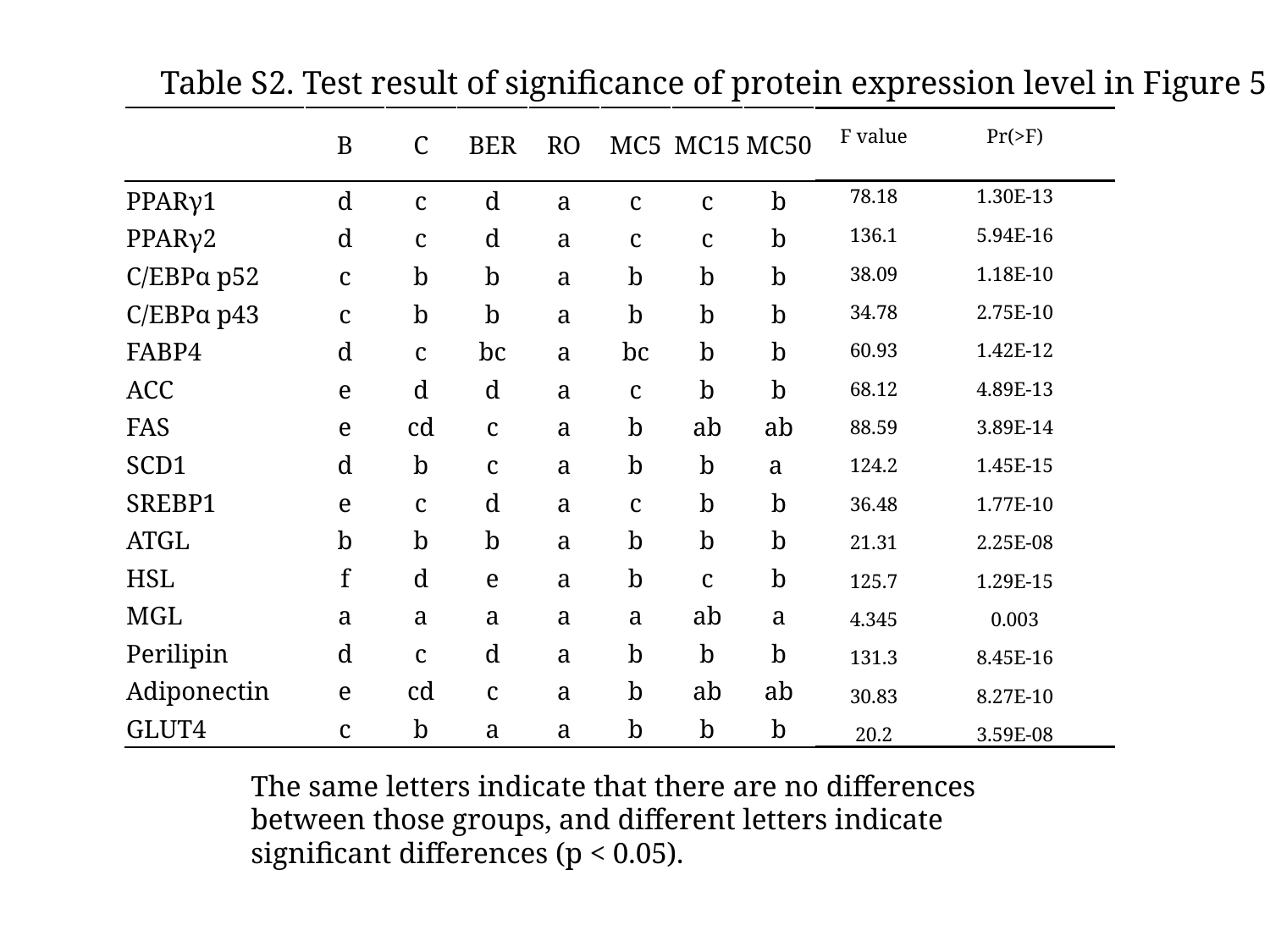

Table S2. Test result of significance of protein expression level in Figure 5
| | B | C | BER | RO | MC5 | MC15 | MC50 |
| --- | --- | --- | --- | --- | --- | --- | --- |
| PPARγ1 | d | c | d | a | c | c | b |
| PPARγ2 | d | c | d | a | c | c | b |
| C/EBPα p52 | c | b | b | a | b | b | b |
| C/EBPα p43 | c | b | b | a | b | b | b |
| FABP4 | d | c | bc | a | bc | b | b |
| ACC | e | d | d | a | c | b | b |
| FAS | e | cd | c | a | b | ab | ab |
| SCD1 | d | b | c | a | b | b | a |
| SREBP1 | e | c | d | a | c | b | b |
| ATGL | b | b | b | a | b | b | b |
| HSL | f | d | e | a | b | c | b |
| MGL | a | a | a | a | a | ab | a |
| Perilipin | d | c | d | a | b | b | b |
| Adiponectin | e | cd | c | a | b | ab | ab |
| GLUT4 | c | b | a | a | b | b | b |
| F value | Pr(>F) |
| --- | --- |
| 78.18 | 1.30E-13 |
| 136.1 | 5.94E-16 |
| 38.09 | 1.18E-10 |
| 34.78 | 2.75E-10 |
| 60.93 | 1.42E-12 |
| 68.12 | 4.89E-13 |
| 88.59 | 3.89E-14 |
| 124.2 | 1.45E-15 |
| 36.48 | 1.77E-10 |
| 21.31 | 2.25E-08 |
| 125.7 | 1.29E-15 |
| 4.345 | 0.003 |
| 131.3 | 8.45E-16 |
| 30.83 | 8.27E-10 |
| 20.2 | 3.59E-08 |
The same letters indicate that there are no differences between those groups, and different letters indicate significant differences (p < 0.05).

## Slide 7
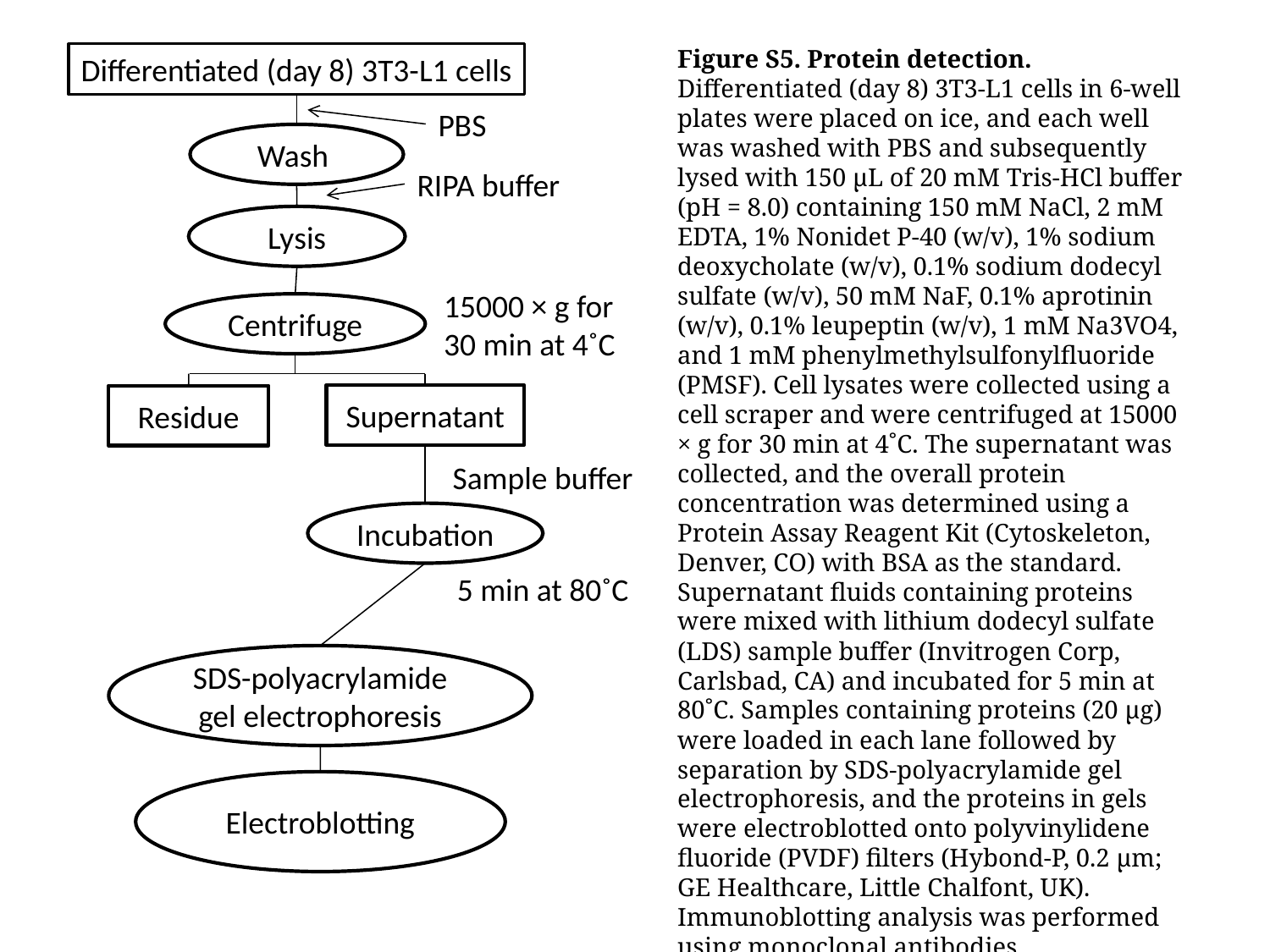

Figure S5. Protein detection.
Differentiated (day 8) 3T3-L1 cells in 6-well plates were placed on ice, and each well was washed with PBS and subsequently lysed with 150 μL of 20 mM Tris-HCl buffer (pH = 8.0) containing 150 mM NaCl, 2 mM EDTA, 1% Nonidet P-40 (w/v), 1% sodium deoxycholate (w/v), 0.1% sodium dodecyl sulfate (w/v), 50 mM NaF, 0.1% aprotinin (w/v), 0.1% leupeptin (w/v), 1 mM Na3VO4, and 1 mM phenylmethylsulfonylfluoride (PMSF). Cell lysates were collected using a cell scraper and were centrifuged at 15000 × g for 30 min at 4˚C. The supernatant was collected, and the overall protein concentration was determined using a Protein Assay Reagent Kit (Cytoskeleton, Denver, CO) with BSA as the standard. Supernatant fluids containing proteins were mixed with lithium dodecyl sulfate (LDS) sample buffer (Invitrogen Corp, Carlsbad, CA) and incubated for 5 min at 80˚C. Samples containing proteins (20 μg) were loaded in each lane followed by separation by SDS-polyacrylamide gel electrophoresis, and the proteins in gels were electroblotted onto polyvinylidene fluoride (PVDF) filters (Hybond-P, 0.2 μm; GE Healthcare, Little Chalfont, UK). Immunoblotting analysis was performed using monoclonal antibodies.
Differentiated (day 8) 3T3-L1 cells
PBS
Wash
RIPA buffer
Lysis
15000 × g for
30 min at 4˚C
Centrifuge
Supernatant
Residue
Sample buffer
Incubation
5 min at 80˚C
SDS-polyacrylamide gel electrophoresis
Electroblotting

## Slide 8
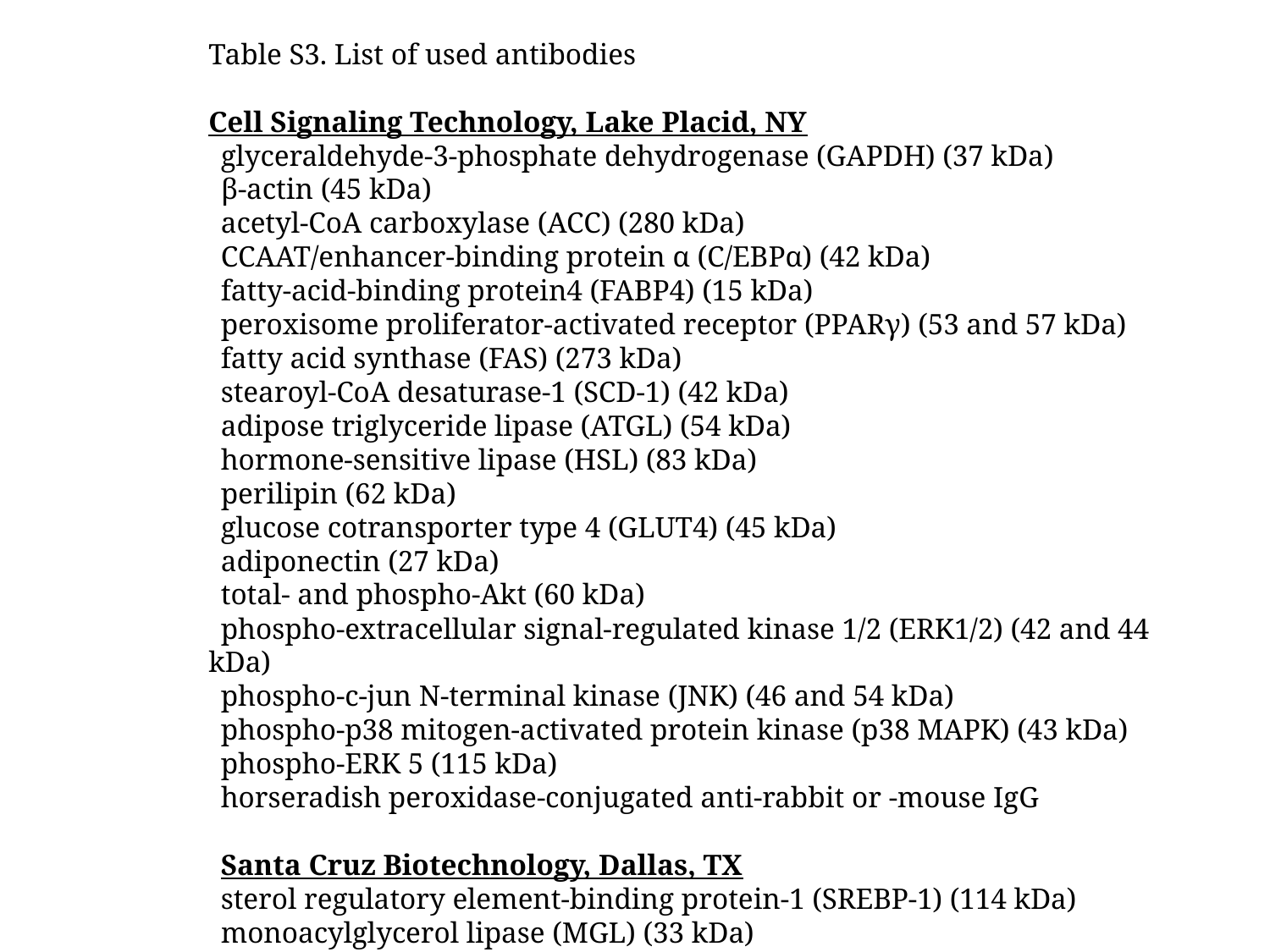

Table S3. List of used antibodies
Cell Signaling Technology, Lake Placid, NY
glyceraldehyde-3-phosphate dehydrogenase (GAPDH) (37 kDa)
β-actin (45 kDa)
acetyl-CoA carboxylase (ACC) (280 kDa)
CCAAT/enhancer-binding protein α (C/EBPα) (42 kDa)
fatty-acid-binding protein4 (FABP4) (15 kDa)
peroxisome proliferator-activated receptor (PPARγ) (53 and 57 kDa)
fatty acid synthase (FAS) (273 kDa)
stearoyl-CoA desaturase-1 (SCD-1) (42 kDa)
adipose triglyceride lipase (ATGL) (54 kDa)
hormone-sensitive lipase (HSL) (83 kDa)
perilipin (62 kDa)
glucose cotransporter type 4 (GLUT4) (45 kDa)
adiponectin (27 kDa)
total- and phospho-Akt (60 kDa)
phospho-extracellular signal-regulated kinase 1/2 (ERK1/2) (42 and 44 kDa)
phospho-c-jun N-terminal kinase (JNK) (46 and 54 kDa)
phospho-p38 mitogen-activated protein kinase (p38 MAPK) (43 kDa)
phospho-ERK 5 (115 kDa)
horseradish peroxidase-conjugated anti-rabbit or -mouse IgG
Santa Cruz Biotechnology, Dallas, TX
sterol regulatory element-binding protein-1 (SREBP-1) (114 kDa)
monoacylglycerol lipase (MGL) (33 kDa)

## Slide 9
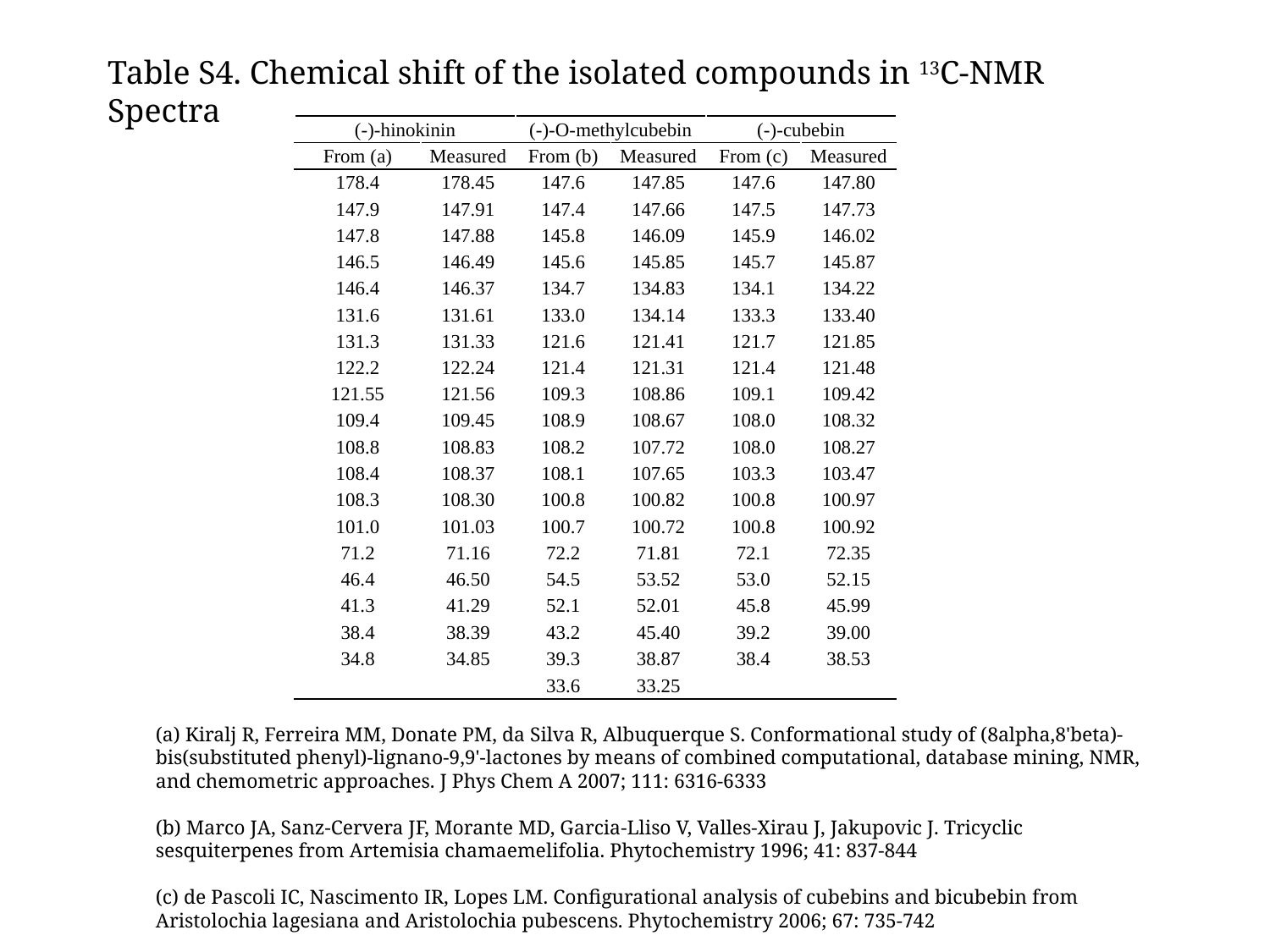

Table S4. Chemical shift of the isolated compounds in 13C-NMR Spectra
| (-)-hinokinin | | (-)-O-methylcubebin | | (-)-cubebin | |
| --- | --- | --- | --- | --- | --- |
| From (a) | Measured | From (b) | Measured | From (c) | Measured |
| 178.4 | 178.45 | 147.6 | 147.85 | 147.6 | 147.80 |
| 147.9 | 147.91 | 147.4 | 147.66 | 147.5 | 147.73 |
| 147.8 | 147.88 | 145.8 | 146.09 | 145.9 | 146.02 |
| 146.5 | 146.49 | 145.6 | 145.85 | 145.7 | 145.87 |
| 146.4 | 146.37 | 134.7 | 134.83 | 134.1 | 134.22 |
| 131.6 | 131.61 | 133.0 | 134.14 | 133.3 | 133.40 |
| 131.3 | 131.33 | 121.6 | 121.41 | 121.7 | 121.85 |
| 122.2 | 122.24 | 121.4 | 121.31 | 121.4 | 121.48 |
| 121.55 | 121.56 | 109.3 | 108.86 | 109.1 | 109.42 |
| 109.4 | 109.45 | 108.9 | 108.67 | 108.0 | 108.32 |
| 108.8 | 108.83 | 108.2 | 107.72 | 108.0 | 108.27 |
| 108.4 | 108.37 | 108.1 | 107.65 | 103.3 | 103.47 |
| 108.3 | 108.30 | 100.8 | 100.82 | 100.8 | 100.97 |
| 101.0 | 101.03 | 100.7 | 100.72 | 100.8 | 100.92 |
| 71.2 | 71.16 | 72.2 | 71.81 | 72.1 | 72.35 |
| 46.4 | 46.50 | 54.5 | 53.52 | 53.0 | 52.15 |
| 41.3 | 41.29 | 52.1 | 52.01 | 45.8 | 45.99 |
| 38.4 | 38.39 | 43.2 | 45.40 | 39.2 | 39.00 |
| 34.8 | 34.85 | 39.3 | 38.87 | 38.4 | 38.53 |
| | | 33.6 | 33.25 | | |
(a) Kiralj R, Ferreira MM, Donate PM, da Silva R, Albuquerque S. Conformational study of (8alpha,8'beta)-bis(substituted phenyl)-lignano-9,9'-lactones by means of combined computational, database mining, NMR, and chemometric approaches. J Phys Chem A 2007; 111: 6316-6333
(b) Marco JA, Sanz-Cervera JF, Morante MD, Garcia-Lliso V, Valles-Xirau J, Jakupovic J. Tricyclic sesquiterpenes from Artemisia chamaemelifolia. Phytochemistry 1996; 41: 837-844
(c) de Pascoli IC, Nascimento IR, Lopes LM. Configurational analysis of cubebins and bicubebin from Aristolochia lagesiana and Aristolochia pubescens. Phytochemistry 2006; 67: 735-742
